# Feasibility and patient acceptability of a commercially available wearable and a smart phone application in identification of motor states in parkinson’s disease

**DOI:** 10.1371/journal.pdig.0000225

**Published:** 2023-04-07

**Authors:** Sammeli Liikkanen, Janne Sinkkonen, Joni Suorsa, Valtteri Kaasinen, Eero Pekkonen, Mikko Kärppä, Filip Scheperjans, Teppo Huttunen, Toni Sarapohja, Ullamari Pesonen, Mikko Kuoppamäki, Tapani Keränen

**Affiliations:** 1 Orion Corporation, Orion Pharma, R&D, Espoo Finland; 2 DRDP, Institute of Biomedicine, University of Turku, Turku Finland; 3 Reaktor Innovations Oy, Helsinki Finland; 4 Clinical Neurosciences, University of Turku, Turku, Finland; 5 Neurocenter, Turku University Hospital, Turku, Finland; 6 Department of Neurology, Helsinki University Hospital and Department of Clinical Neurosciences (Neurology), University of Helsinki, Helsinki, Finland; 7 Department of neurology, Oulu University Hospital, Oulu Finland; 8 Estimates Oy, Turku Finland; 9 Institute of Biomedicine, University of Turku, Turku Finland; 10 Institute of Clinical Medicine, University of Eastern Finland, Kuopio Finland; University of Cagliari: Universita degli Studi Di Cagliari, ITALY

## Abstract

In the quantification of symptoms of Parkinson’s disease (PD), healthcare professional assessments, patient reported outcomes (PRO), and medical device grade wearables are currently used. Recently, also commercially available smartphones and wearable devices have been actively researched in the detection of PD symptoms. The continuous, longitudinal, and automated detection of motor and especially non-motor symptoms with these devices is still a challenge that requires more research. The data collected from everyday life can be noisy and frequently contains artefacts, and novel detection methods and algorithms are therefore needed. 42 PD patients and 23 control subjects were monitored with Garmin Vivosmart 4 wearable device and asked to fill a symptom and medication diary with a mobile application, at home, for about four weeks. Subsequent analyses are based on continuous accelerometer data from the device. Accelerometer data from the Levodopa Response Study (MJFFd) were reanalyzed, with symptoms quantified with linear spectral models trained on expert evaluations present in the data. Variational autoencoders (VAE) were trained on both our study accelerometer data and on MJFFd to detect movement states (e.g., walking, standing). A total of 7590 self-reported symptoms were recorded during the study. 88.9% (32/36) of PD patients, 80.0% (4/5) of DBS PD patients and 95.5% (21/22) of control subjects reported that using the wearable device was very easy or easy. Recording a symptom at the time of the event was assessed as very easy or easy by 70.1% (29/41) of subjects with PD. Aggregated spectrograms of the collected accelerometer data show relative attenuation of low (<5Hz) frequencies in patients. Similar spectral patterns also separate symptom periods from immediately adjacent non-symptomatic periods. Discriminative power of linear models to separate symptoms from adjacent periods is weak, but aggregates show partial separability of patients vs. controls. The analysis reveals differential symptom detectability across movement tasks, motivating the third part of the study. VAEs trained on either dataset produced embedding from which movement states in MJFFd could be predicted. A VAE model was able to detect the movement states. Thus, a pre-detection of these states with a VAE from accelerometer data with good S/N ratio, and subsequent quantification of PD symptoms is a feasible strategy. The usability of the data collection method is important to enable the collection of self-reported symptom data by PD patients. Finally, the usability of the data collection method is important to enable the collection of self-reported symptom data by PD patients.

## Introduction

Parkinson’s disease (PD) is one of the most common neurodegenerative disorders with a prevalence of 1–2 per 1000 persons, rising with age [1]. The disease primarily affects dopaminergic neurons in a specific brain area, substantia nigra, leading to typical motor symptoms [2]. Patients with PD suffer also from non-motor symptoms such as constipation, orthostatic hypotension, depression, anxiety and cognitive problems, which may even precede motor symptoms [2]. As disease-modifying treatments are still waiting to be discovered, understanding the subjective nature of the disease and how the person perceives his/her symptoms, as well as how motor and non-motor symptoms are associated with the current therapies in PD, is vital to effectively personalise the treatment [3].

Most of the patients with PD are treated with the dopamine precursor levodopa (LD) [4]. With the progression of PD, patients begin to experience wearing-off symptoms, i.e., fluctuations in tremor and mobility as well as appearance of involuntary movements (dyskinesia), due to variation in LD plasma concentrations [4]. Patients with advanced LD-treated disease may experience rapid motor symptom exacerbations, i.e., ON-OFF phenomena [5].

Various scale and diary tools have been developed for the assessment and recording of PD symptoms, but these tools have certain limitations and may be an extra burden to patients [6,7]. Assessment scales are liable to subjective interpretations and reliability of diary data may be compromised by deficient memory functions of the patients [6,8]. Continuous monitoring of both motor and non-motor symptoms in patients with PD would offer an opportunity to improve therapeutic regimens in both standard care and especially in clinical trials where accuracy in valuation of treatment effects is critical [9]. Both patients and health care professionals see promises in wearable monitoring technologies in supplementing and improving the care of PD [8,10]. Several solutions for continuous monitoring of PD have been presented [11,12]. Many of the technologies require use of several sensors attached to the body [11–13]. However, patients with PD prefer small, easy-to-use devices that would not interfere with their daily routine [8,10]. Several studies have evaluated the feasibility and accuracy of data collected using a smartwatch and smartphone [14–16]

A substantive literature on detection of Parkinson symptoms from wearable data exists [17,18]. In the present study, we explored whether use of a commercially available wearable device together with a mobile application allows capturing data that would further enable reliable assessment of motor fluctuations and dyskinesia in PD patients. The primary objective of the study was identification of OFF symptoms, as perceived by the subject, with reasonable accuracy from real-world data collected with commercially available Garmin Vivosmart 4 wearable device and a mobile application.

As our own data was collected in the real-world settings and thus was noisy, and the population size was limited, we also acquired and re-analysed a dataset (“MJFFd”) from the Levodopa Response Study, funded by the Michael J. Fox Foundation [16]. From MJFFd, we found that it is easier to spot symptoms during specific motor activities. This is because the patient’s movement state during the task affects symptom detection. To address this, we trained a deep-learning model, VAE, to condense information from accelerometer signals [19]. To check if the VAE was effective, we used it to predict the motor task being performed from the representation it generated from the accelerometer data. The accuracy of the VAE’s predictions suggests that it is capable of capturing meaningful movement states. VAEs or similar unsupervised models could therefore be useful in future to detect movement states where symptoms are prominent.

## Material and methods

### Study description

This was an open, non-randomized, real-world data study. Patients fulfilling the diagnostic criteria for PD [20], receiving LD treatment, and with a history of motor fluctuations, i.e., daily LD-treatment related changes in the severity of tremor, bradykinesia and/or rigidity were included. Six patients had advanced PD had Deep Brain Stimulation (DBS) devices. Two patients had Activa PC (Medtronic) and four St. Jude Medical Infinity (Abbott) electrodes. The participants were recruited at outpatient clinics of Helsinki University Hospital, Oulu University Hospital and Turku University Hospital by neurologists with expertise in PD. Spouses of the PD patients, free of any neurological disorder, were invited as control subjects.

All the subjects gave their written informed consent for voluntary participation in the study. The study was approved (R19051 June 4th, 2019) by the Research Ethics Committee of the Pirkanmaa Hospital District, Finland.

**Fig 1:**
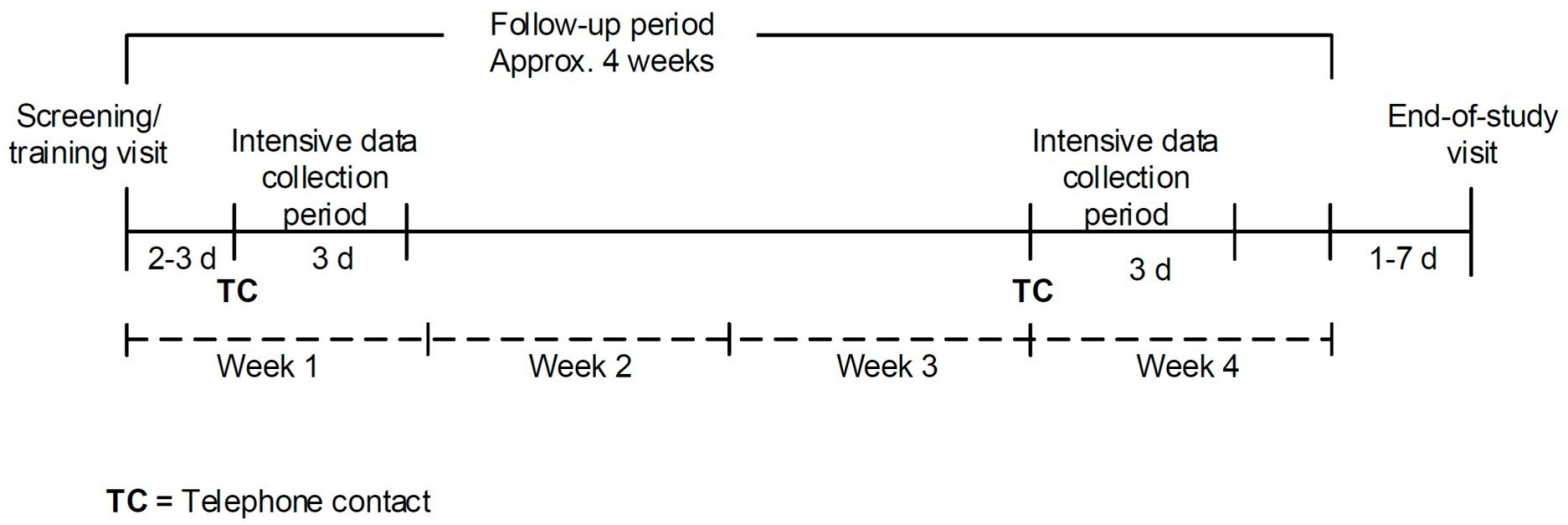
The Study design.

There were two visits to the study sites during the study. The first visit was a combined screening and training visit. The screening (Baseline characteristics, Hoehn and Yahr stage, and Unified Parkinson’s-disease rating scale (UPDRS parts I-IV)) was performed by an investigator and the training was provided by a study nurse. After this visit, the study subjects’ everyday life was followed for approximately four weeks with a wearable device. This follow-up period included two 3-day intensive data-collection periods, when subjects were specifically requested to report about all their symptoms very accurately. The second visit to the study centre (end-of-study visit) took place within 7 days after completion of the follow-up period. At the end-of-study visit, UPDRS II (Self-evaluation of activities of daily living) scale was assessed for the PD patients by the study nurse. In addition, a usability questionnaire was completed for all subjects by the study nurse who also interviewed the subject. The study design is presented in Fig 1.

The study intervention was Garmin Vivosmart 4 (Firmware versions were v3.10, v4.00, v4.10 and v4.20) as automatically updated by the vendor during the study period. However, as the data analysis of movement data was based on the raw data accessed straight from the accelerometer sensor, these updates had no impact on the results and a bespoke Android mobile application specifically designed and built for this this study (Xiaomi mobile phones with Android v8.10). The application included three key functionalities:

1. a medication reminder feature for levodopa and other PD related medication schedule, that was set up together with the study nurse at screening/training visit,
2. symptom collection feature with which the subject was able to record motor and non-motor symptoms, both during the symptoms and/or retrospectively, and
3. automatic transfer of the Garmin wearable data (and patient reported data from mobile app to server via mobile network.

**Fig 2:**
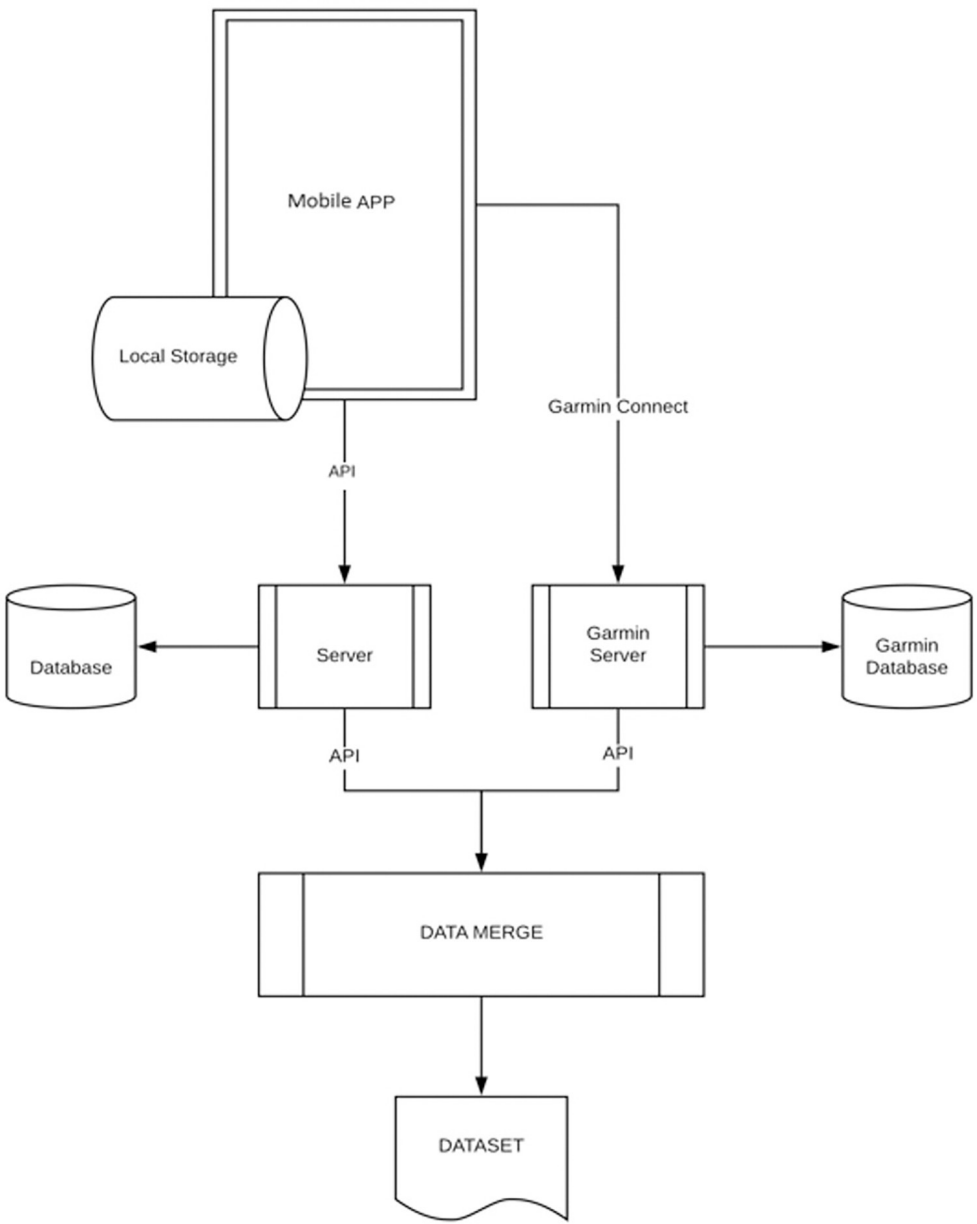
The data collection architecture of the Garmin wearable and the mobile app.

**Fig 3:**
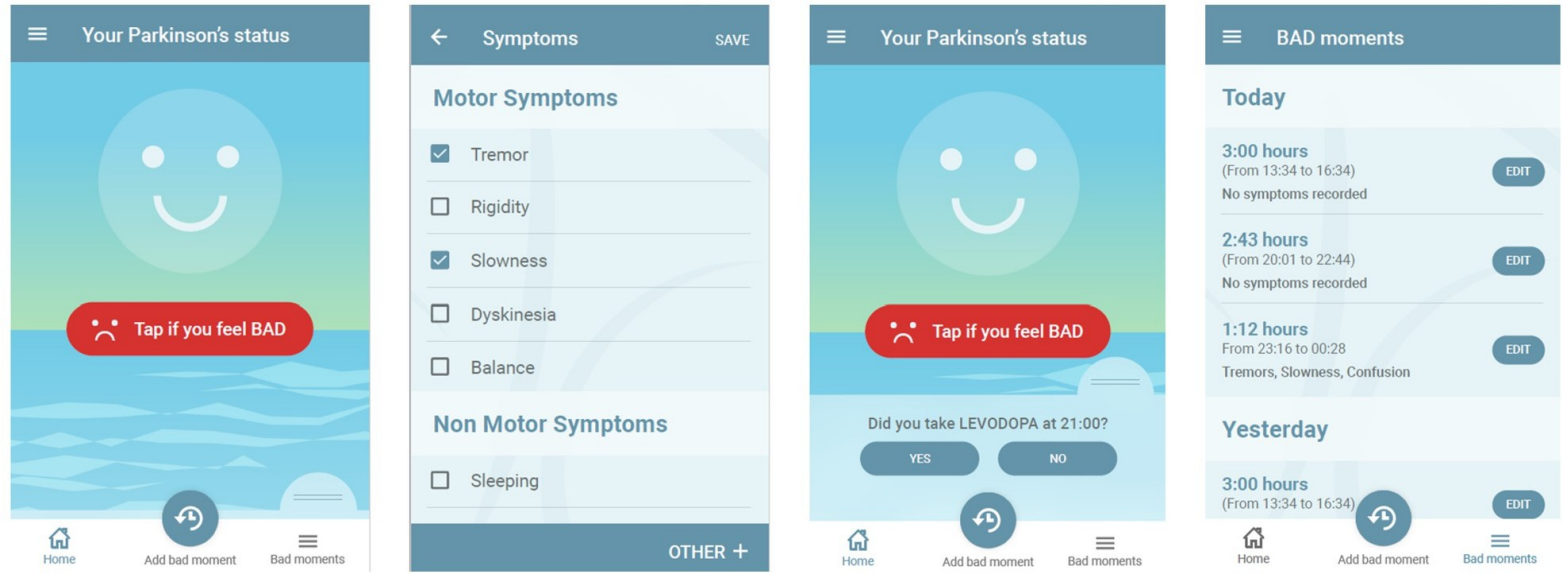
Examples of the mobile app features (from left to right: the main page, adding of symptoms, medication reminder and list of reported symptoms).

Both Garmin wearable and the mobile app were set to use the same clock server to ensure accurate labelling of the data by a subject. Accelerometer raw data was sampling rate was 50Hz, heart rate $1\frac{1}{3}$ Hz, and heart rate variability $1\frac{1}{3}$ Hz. The high-level data collection architecture of wearable and the mobile app is described in Fig 2, and example screens of mobile are shown in Fig 3.

The total numbers of subjects planned was 50 (30 subjects with PD and 20 control subjects without PD). The actual number of enrolled subjects was 65 (42 subjects with PD and 23 control subjects without PD). The difference was explained by high interest for participation. Of the 65 subjects, 3 subjects (all PD patients) had little to no real-world data to be used in the analysis because of premature discontinuation (2 subjects) or technical data transfer issues (1 subject). The data from these subjects were not used in analyses.

### Statistical methods

Demographic and baseline characteristics as well as all applicable efficacy and safety data were summarised using descriptive statistics. No formal statistical hypotheses were specified for this study. The number of participants was based on assumption of events required for distinction of subjects with PD and control subjects. No formal sample size calculation was used. Wearable and mobile-device data collected from subjects with PD and without PD during the follow-up period were analysed by using exploratory data analysis with R and Stan.

### Symptom diary data

During the follow-up period, subjects’ everyday life, as well as motor and non-motor data were measured and collected with the sensors of commercially available Garmin Vivosmart 4 wearable device from all the subjects. Levodopa treatment intake and subjective symptom data were collected with a mobile device application from subjects with PD. The symptoms were collected by allowing a subject to report “a bad moment” by pushing one specific button designed to be easily accessible within the front page of the mobile application. Once a symptom had finished, the subject was accordingly able to end the symptom reporting by similar one button in the front page. After this, subject was asked to classify this symptom by either choosing the correct symptom from a pre-defined list (*tremor*, *rigidity*, *slowness*, *dyskinesia*, *balance*, *sleeping disturbances*, *anxiety*, *dizziness*, *hallucination*, *symptom of smelling or tasting*, *symptom of urination*) built into the application or as free text under the heading ‘other’. In the analysis, symptoms were divided into tremor and other movement symptoms. Totally there were sensor data from 578 tremor events and 2253 events with other symptoms.

### Accelerometer data

Only the data from the three-axis accelerometer of the wearable device were used. With its recording rate of 50 Hz, blocks of 65536 samples (21.8 minutes each), interleaved for double cover, were analyzed spectrally. The time scale of 21.8 minutes was chosen to be like the time scale of the reported symptoms in diaries, which typically was on the order of tens of minutes. Blocks with apparent sampling problems, as discovered by time stamps of the samples, were discarded. In total, recordings from 62 subjects covered 191,411 blocks of which 169,627 (total of 88% were accepted).

### Spectral pre-processing pipeline

Spectral processing included Fast Fourier Transform (FFT), and subsequent calculation of logarithmic, dB-scale power spectra at 10–15 bins depending on analysis, logarithmically spaced. In addition to the original channels (x, y, z), we calculated auxiliary, derived channels. Neither of these virtual channel sets provided markedly improved S/N ratio compared to the original, but for completeness, we describe them here, and they also appear in the later analyses.

The first set of derived channels was aligned to an estimate of gravity (g), and its temporal differentials (here called dg, ddg), which are orthogonal to g. Lacking gyroscopes, gravity can only be estimated more or less poorly. Our estimate of g was obtained by (causal) 0.1Hz low-pass filtering; this gives a good approximation when the subject is still, otherwise not. This gives us another, moving coordinate system for the measurements. The aim, however, was just to get another, potentially useful, moving coordinate system for the measurements.

The second set of derived channels (*-PC1, *-PC2) was obtained by first taking an orthogonal channel triplet (either of the two above), then after FFT finding, for each frequency and sample, the two principal axes of oscillation described by the frequency-wise three complex numbers and measuring amplitude of these oscillations. Technically, this is an eigenvalue decomposition of a matrix. Attractively, these two derived channels are invariant to (fixed) rotations of the device coordinate system.

Discriminative models in this paper are linear and based on the logarithmic (dB-scale) power spectra of the accelerometer signal.

### Characterization and separability of symptom events

Detection of symptoms from the accelerometer signal can only be accurately assessed with a ground truth for comparison. In contrast to the MJFF study, where third-party assessment of symptoms is available in a temporally accurate form, our ground truth is based on patient diary entries. These entries may not always be recorded during the actual occurrence of symptoms, and the reported durations may sometimes be longer than the actual duration of the symptoms. As a result, we chose to synchronize our analysis to the middle of the reported symptom period, and we rejected symptoms with reported durations longer than two hours.

**Fig 4:**
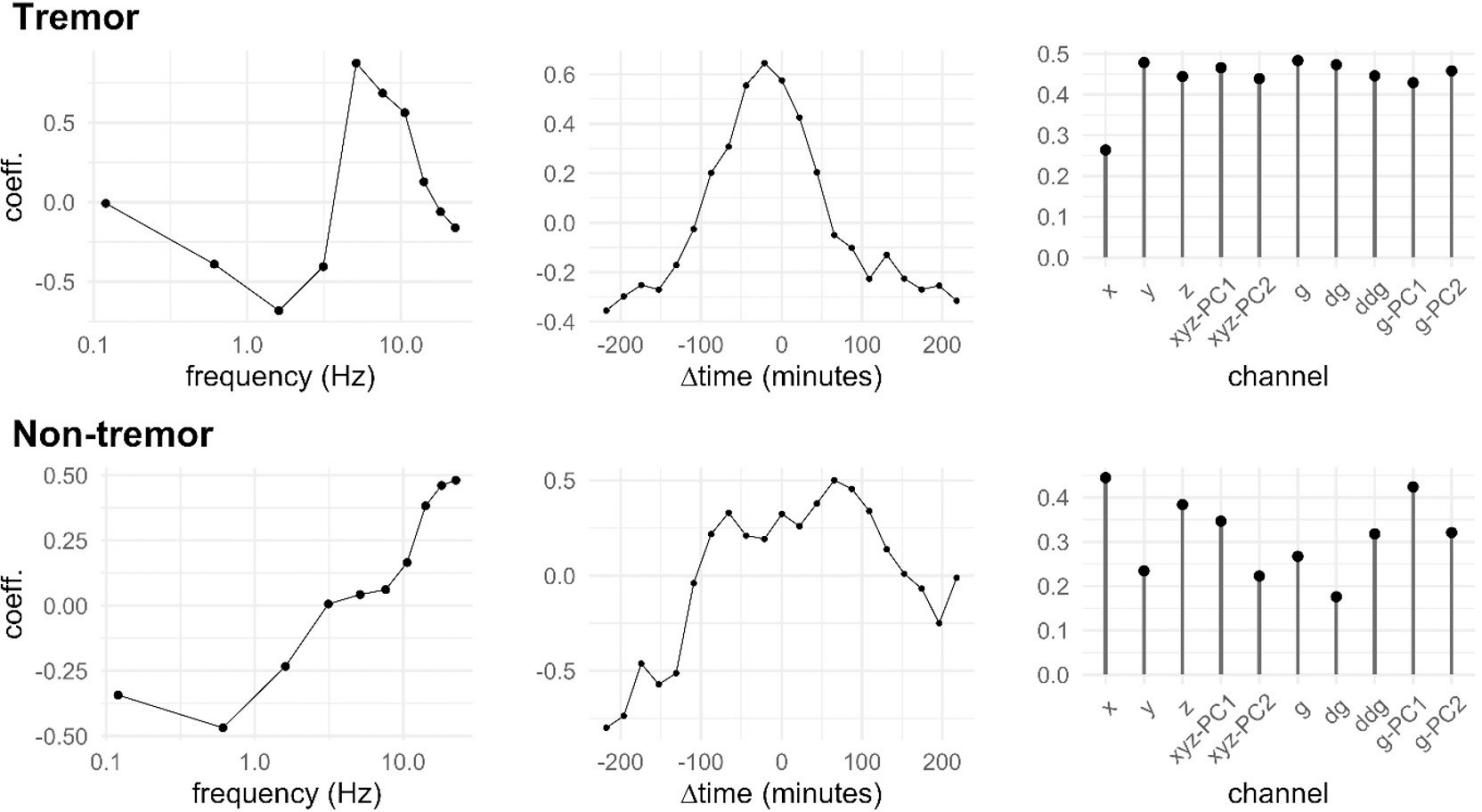
Spectral, temporal, and across-channel envelopes of tremor symptom data (top) and rest of the symptoms data (bottom).

To characterize the average strength of symptoms (indexed here by i, with subject identities ignored) around the central timestamp (at relative time t), and across frequencies (f) and channels (c), we used a factorial model, for tremor and other symptoms separately. As its output, such a model produces three envelopes (separately for both symptom types; see Fig 4): temporal shape, spectral shape, and strength across channels. If we denote the logarithmic amplitude of symptom i by $L^{i}tfc$, the model is simply $L^{i}tfc∼N(u_{t}v_{f}w_{c}+b,\sigma^{2})$, where the coefficients u, v, and w characterize the temporal, frequency, and channel shape of the symptom, respectively, b is an intercept (baseline), and σ^2^ the error variance. The model was fitted with maximum likelihood, equivalent to least squares in this case, using the optimization feature of Stan.

### Methods with the MJFF dataset

The MJFF Levodopa-Response study dataset contains accelerometer measurements from 30 participants, with and without levodopa infusion (“drug state” in later discussion), in different tasks, and some repetitions within the task. For each of these replicates, or combinations of the afore-mentioned variables, tremor, bradykinesia, and dyskinesia are scored by external observers onto an ordinal scale. Of all available data, we used only accelerations from the dominant hand, and the observer scores.

To get scalar measures of symptoms for replicates as seen in the accelerometer data, we trained ordinal (linear) regression models for each symptom, on spectral features of the accelerometer data. The models were L2-regularized with the regularization coefficients cross-validated. This left us with three models, one for each symptom type (tremor, bradykinesia, dyskinesia). The replicates were then scored with the models to get an indicator of symptoms (“accelerometer score”), parallel to the observer scores, that is a function of accelerometer data only.

Spectral analysis pipeline for the MJFF data was mostly identical to that applied to our own data, described above. All the three original accelerometer channels were used, with the three derived “g-channels” (g, dg, ddg), all with eleven frequency bins with logarithmic placements, for logarithmic, dB-scale spectral-power features.

With the observer and accelerometer scores available, we studied how symptoms, as defined by those scores, are affected by the drug state and task, with the subject-level variation controlled. For this, we modelled the scores with Bayesian linear models, the models having random-effect terms for various levels of replications (subject, task, drug state, session number within visit). The model for accelerometer scores was with multinormal residuals (all symptoms combined into the same model). The observer models were with ordinal logistic (and separate because the bradykinetic scores had lots of missing values). The models were fitted with Stan using the R package brms [21].

### Training variational autoencoders (VAE)

A common approach in supervised learning situations where labelled data is expensive to produce, but unlabelled is cheap, is to combine unsupervised and supervised learning. The unsupervised learning part typically uses a large collection of data relevant to the task, but not specifically labelled for it. A suitable model is chosen that can learn general representations of the unlabelled data, which are then used as input in a downstream predictive task with task-specific labelled data.

To test whether a VAE can create useful representations of movement states (different motor tasks in MJFFd) from accelerometer data, we used a subset of the MJFFd data to first train a VAE, and then used the trained VAE to create representations for accelerometer data with the task of predicting which of the predetermined tasks the patient was performing.

## Results

### Background variables

Study consisted of 42 PD patients and 23 controls. Six of the PD patients had DBS (DBS PD) devices. Overall, the general demographic and baseline characteristics were similar between PD patients and controls (Table 1).

**Table 1:** Demographics of the study population.

Variable		Subjects with PD (N = 42)			Control subjects (N = 23)
		*PD patient (N = 36)*	*DBS PD (N = 6)*		
**Gender**					
*Male*	n	15 (41.7%)	1 (16.7%)		11 (47.8%)
*Female*	n	21 (58.3%)	5 (83.3%)		12 (52.2%)
**Age (years)**	range	43–75	48–69		46–74
	mean (SD)	63.4 (7.8)	61.3 (8.3)		64.9 (7.9)
	median	63.0	63.5		67.0
**Weight (kg)**	range	46.2–124.4	53.5–136.0		54.0–120.0
	mean (SD)	79.72 (18.20)	91.07 (28.35)		81.98 (18.75)
	median	75.70	85.75		82.40
**Height (cm)**	range	152.0–190.5	162.0–177.0		154.0–185.0
	mean (SD)	170.27 (11.04)	167.83 (5.64)		168.98 (8.53)
	median	170.00	168.00		170.00
**BMI (kg/m^2)**	range	17.4–46.3	20.4–47.1		20.7–40.1
	mean (SD)	27.58 (6.23)	32.07 (8.85)		28.64 (5.85)
	median	26.85	31.30		26.50
***Variable***		***Subjects with PD (N = 42)***			
		***PD patient (N = 36)***		***DBS PD (N = 6)***	
**Years since the symptoms started**	range	3–22		8–17	
	mean (SD)	10.7 (4.8)		12.3 (2.9)	
	median	11.0		12.5	
**Years since the diagnosis of PD**	range	0–18		5–17	
	mean (SD)	8.5 (4.7)		11.2 (4.0)	
	median	8.5		11.5	
**Years since levodopa treatment started**	range	0–13		3–17	
	mean (SD)	5.3 (3.5)		8.2 (4.8)	
	median	4.0		7.5	
**Years since the end-of-dose wearing off symptoms started**	range	0–7		2–6	
	mean (SD)	2.1 (1.9)		4.0 (1.9)	
	median	1.0		4.0	
**Number of daily levodopa doses**	range	4–12		5–7	
	mean (SD)	5.7 (1.9)		6.0 (1.0)	
	median	5.0		6.0	
**Total daily levodopa doses (mg)**	range	150–2275		250–700	
	mean (SD)	611.8 (343.7)		440.0 (171.0)	
	median	562.5		400.0	
**Hoehn and Yahr Stage**					
*Stage 2*.*0*	n (%)	8 (22.2)		2 (33.3)	
*Stage 2*.*5*	n (%)	10 (27.8)		4 (66.7)	
*Stage 3*.*0*	n (%)	18 (50.0)		0 (0.0)	
**UPDRS total scores at screening**					
*I*. *Mentation*, *behavior and mood*	mean (SD)	1.8 (1.3)		1.2 (1.0)	
*II*. *Activities of daily living*	mean (SD)	12.2 (4.7)		10.8 (6.0)	
*III*. *Motor examination*	mean (SD)	25.4 (10.7)		14.0 (8.6)	
*IV*. *Complications of therapy*	mean (SD)	6.6 (2.7)		4.5 (2.7)	

All DBS PD subjects except one were on levodopa medication at screening visit. Of the 42 subjects with PD (including DBS PD), 34 subjects were also treated with other medications for PD in addition to levodopa, dopamine agonists (67% of patients) and monoamine oxidase B inhibitors (61% of patients) being clearly the most common classes of these medications.

### Efficacy and usability

The UPDRS section-II total scores for subjects with PD were similar at screening and at the end of study (median score of 12.5 at both time points). 63 (36 PD patients, 5 DBS and 22 control subjects) subjects out of 65 responded to the usability questions, however some subjects did not answer to all questions. Usability assessments can be seen from Table 2, and despite there are things (e.g., charging of devices and recording current bad moment) to improve, the overall usability enabled the wanted study conduct in practice.

**Table 2:** Usability of wearables and mobile application.

	PD patient (n = 36)	DBS PD patient (n = 6)	Control (n = 23)
Question	n (%)	n (%)	n (%)
Difficulty of wearing device entire day: very easy or easy	32/36 (88.9%)	4/5 (80.0%)	21/22 (95.5%)
Difficulty of charging device and phone: somewhat or very difficult	13/36 (36.1%)	2/5 (40.0%)	7/22 (31.8%)
LEVODOPA reminders helpful?	26/35 (74.3%)	3/4 (75.0%)	NA
Recording current bad moment difficulty: very easy or easy	25/36 (69.4%)	4/5 (80.0%)	NA
Recording previous bad moment difficulty: very easy or easy	24/36 (66.7%)	4/5 (80.0%)	NA
Learned more of condition with app?	29/36 (80.6%)	0/5 (0.0%)	NA

### Symptom recording

Of the 42 subjects with PD, 40 (95.2%) recorded a total of 7590 symptoms (motor, non-motor or other) in the application (Table 3). A total of 39 subjects (92.9%) reported 4492 motor symptoms (pre-defined or other), of which the most reported were rigidity (1513 events in 36 subjects [85.7%]), slowness (1327 events in 37 subjects [88.1%]) and tremor (925 events in 30 subjects [71.4%]).

Non-motor symptoms (pre-defined or self-described) were reported at 1502 occasions by 35 subjects (83.3%). The most reported non-motor symptom was pain (893 events in 26 subjects [61.9%]), followed by dizziness (182 events in 16 subjects [38.1%]), urinary dysfunction (177 events in 10 subjects [23.8%]) and sleep disturbances (118 events in 20 subjects [47.6%]).

**Table 3:** Event counts of PD symptoms reported in the mobile application and the relative amount of the subjects who had reported that symptom type at least once.

	PD patient (n = 36)	DBS PD patient (n = 6)
Symptoms	n (% out of total)	n (% out of total)
Motor symptoms, pre-defined	4109 (97.2%)	243 (66.7%)
Non-motor symptoms, pre-defined	786 (80.6%)	33 (83.3%)
Other	2407 (66.7%)	12 (33.3%)
Other symptoms, re-classified motor symptoms^1^	140 (13.9%)	-
Other symptoms, re-classified non-motor symptoms^1^	683 (27.8%)	-

Notes: Grouped here with other symptoms^1^

### Linear models on spectral features see the signal

As a feasibility check, total aggregates of all data show partial separability of patients vs. controls, based on the ratio of low vs. high (cut-off ≈5Hz) frequencies. Similar spectral patterns also separate symptom periods from immediately adjacent non-symptomatic periods.

**Fig 5:**
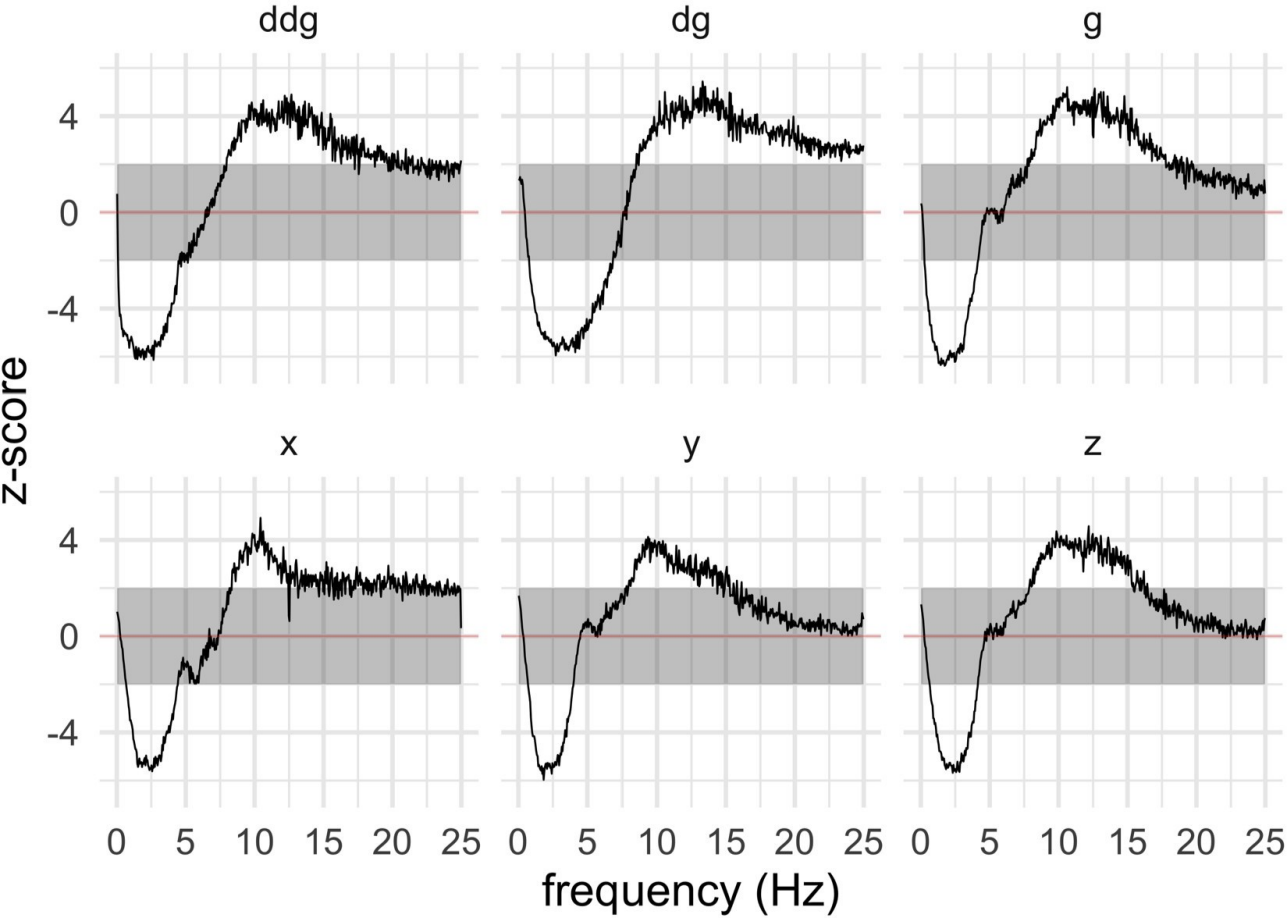
Temporally aggregated power spectra of device and g-aligned channels patients vs. controls.

**Fig 6:**
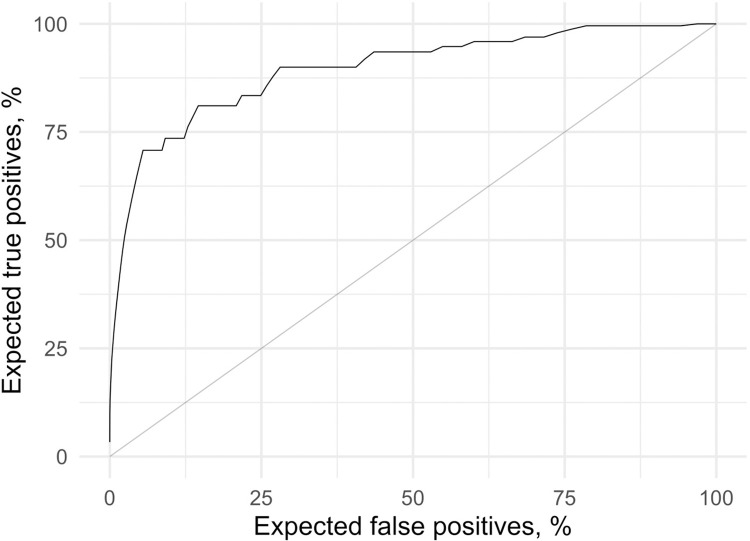
Leave-one-out ROC curve (AUC = 0.895).

To get an idea of spectral effects of the disease, Fig 5 shows spectral differences between patients and controls; z-scores are for block means of dB-scale spectra over all data (and therefore ignore annotations, and also patients as a replication unit). Z-score is calculated as $\frac{\left(mean\_P-mean\_C\right)}{\sqrt{(\frac{var\_P}{n\_P})+\frac{var\_C}{n\_C}}}$, where mean are means of log-amplitudes over the 21.8-minute blocks at the target frequency, for patients (P) and controls (C) respectively, var are variances, and n are numbers of blocks. Next, patients were separated from controls by using the x-channel spectrum. The leave-one-out ROC is shown in Fig 6. Using all available pre-processed wearable data, a promising (AUC = 0.895) classification to patients and controls could be observed, but without annotations separating patients from controls is still incomplete.

With annotations and the factorial model described in the Methods section, one can characterize symptom events of patients with spectral, temporal, and across-channel envelopes as shown in Fig 4. The top row is for tremor symptoms, the bottom row for the rest. The tremor events have a rather striking spectral spike at around 5 Hz, and attenuation at lower frequencies, while other symptoms just show an attenuation of low frequencies. Tremors are temporally more focused around the reported time. Channel responses show varying visibility, with the derived virtual channels (g, dg, etc.) being not markedly worse or better than the physical ones (x, y, z). Tremor is less visible on the x-channel because that channel is parallel to the limb.

### MJFFd analysis suggests the signal visibility depends on the movement states

**Fig 7:**
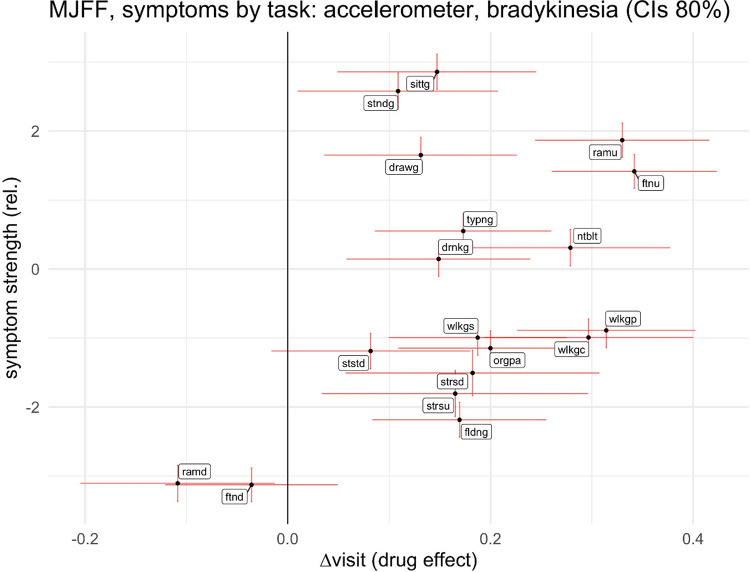
Drug effect on the symptom severity as predicted from the accelerometer data (x-axis), and relative strength of the effect (y-axis at different tasks in MJFFd.

Effect of the drug state on symptoms can be seen in both observer and in accelerometer scores, and with expected polarity, which means dyskinesia is amplified, and bradykinesia and tremor attenuated by the levodopa treatment. We present the effects on bradykinetic accelerometer scores in the Fig 7, the rest can be found in S1 Fig to S3 Fig.

In the figure, task types are depicted by acronyms (again, see S2 Fig for the complete list). X-axis shows the difference from the drug state, and the y-axis the strength of the symptom signal as seen by the linear spectral model. Of these, the effect of levodopa (x) is more meaningful, because it shows the effect of a well-controlled manipulation in a constant movement state. We see clear differences across tasks; especially ramu (repeated arm movement, undominant hand) and ftnu (finger to nose, undominant) are discriminative, also wlkgp (walking through a narrow passage), wlkgc (walking while counting) and ntblt (assembling nuts and bolts). Other symptom types show larger uncertainty in drug effects (S2 Fig and S3 Fig), but notably, ramu and ftnu again show good discriminative ability for dyskinesias. Scales on the axes are commensurable but otherwise artificial.

We did a similar analysis for the observer scores; these are also available as S4 Fig to S6 Fig. Modelling uncertainty there is higher, indicating more noise in observer scores compared to the accelerometer. Partly due to noise, it is hard to say whether symptom visibility across tasks is similar to that with the accelerometer, but discrepancies would not be unexpected, because obviously visual vs. kinetic observability may vary across situations.

In conclusion, these results suggest that even simple linear models are capable of seeing symptom signals from the accelerometer data, and that their capability is modulated by the task, or movement state, of the subject.

### VAE allows separation of movement states

The VAE was implemented using PyTorch. The encoder was a two layer fully connected neural network with node count of 400 and 20 for the first and second layer, a symmetric decoder was used. From the MJFFd we chose a subset of right-handed patients who wore the accelerometer on their right hand. The VAE was trained using 10 second blocks of three channel accelerometer data.

We used the 20-dimensional latent representation of a given block of accelerometer data for a downstream classification task to different movement tasks (S2 Fig). Classification was done by using a fully connected neural network with two hidden layers with 50 nodes each from the scikit-learn library. Classification accuracy was determined by fivefold cross validation. Further validation was done by training the VAE model on patients from one test site and performing cross validated classification using patients from another test site. As a second test, we trained the VAE using our own patient accelerometer data and performed the prediction task on the MJFFd population.

**Fig 8:**
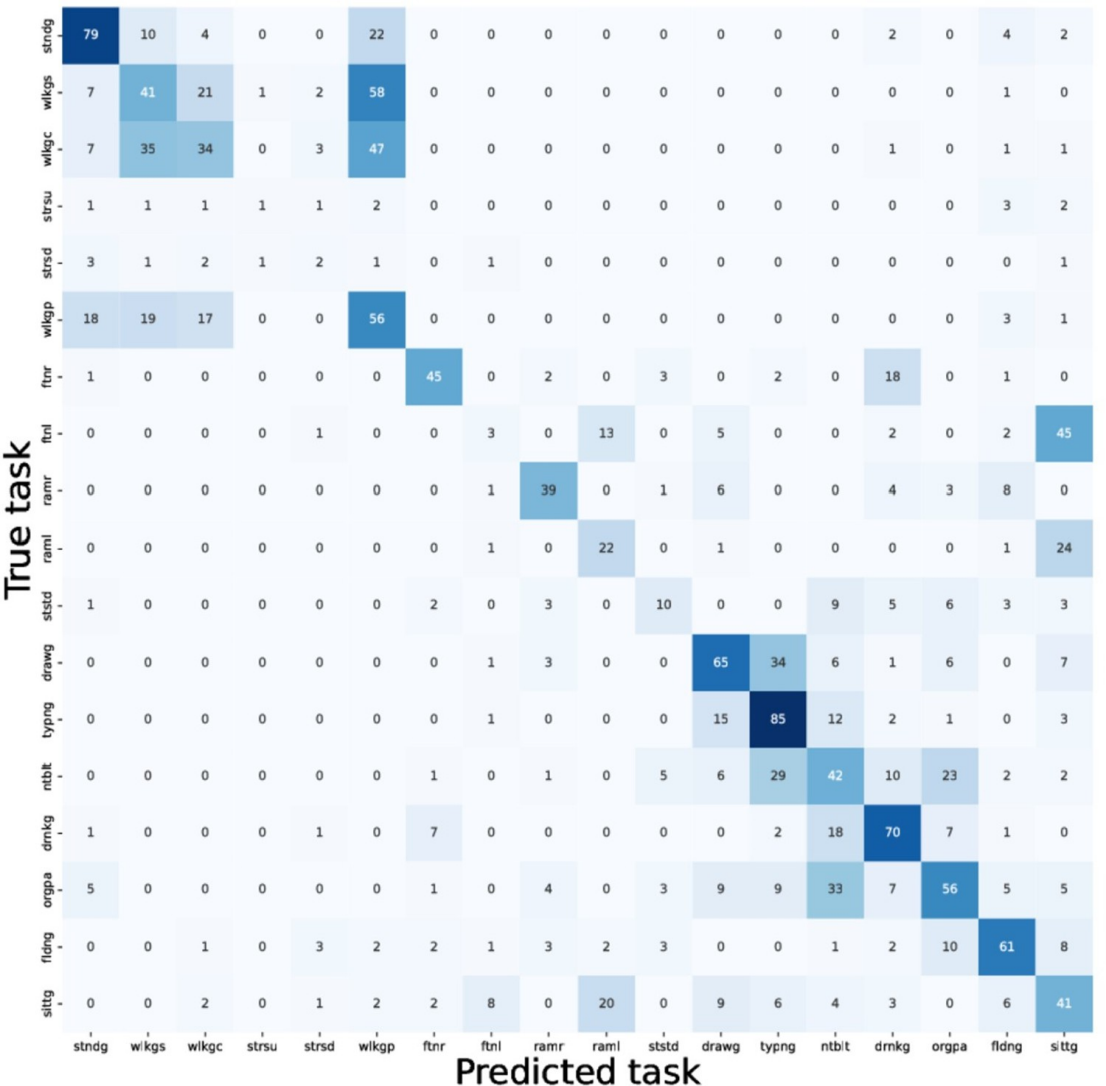
A confusion matrix for predictions of tasks using representations from a VAE trained on MJFFd. Data from Boston site was used for training, and data from NYC site for testing.

When training the model on MJFFd, we obtain a mean classification accuracy of 0.48 for the eighteen different tasks. When the model is trained on our own accelerometer data and tested on MJFF data we obtain a mean classification accuracy of 0.41. Random guessing would give a rough baseline accuracy of approximately 1/18 (~6%). A notable feature of the confusion matrix (Fig 8) is the grouping of confusion among similar tasks: the upper left corner indicates confusion among walking tasks, and the lower right corner shows confusion among fine motor tasks. Since some of the tasks are performed unilaterally, we can also see confusion among tasks where the non-performing hand is approximately stationary: sitting, finger to nose left, and repeated arm movement left.

In both cases, the predictor differentiated the tasks well above random guessing without any tuning, implying the VAE learned meaningful movement-state representations.

## Discussion

In this pilot study, we used a consumer-grade accelerometer and mobile application to monitor symptoms of Parkinson’s disease (PD). From the analysis of Levodopa Response Study data (MJFFd), we also found that movement state can affect the visibility of PD symptoms. Additionally, we discovered that unsupervised variational autoencoders (VAEs) can make it relatively easy to estimate movement states.

The design and usability of the data collection arrangements in this study by using a commercial wearable device and a bespoke mobile application were successful. 80.0% or more of subjects in each cohort assessed wearing the device an entire day or night as very easy or easy. Also, the reminders were considered helpful and the symptom recording easy/very easy by a clear majority of PD patients. The media age of study participants was as low as 63 (43–75) years, which can be seen as a limitation of this study. However, we did not notice any age-related usability difficulties which suggests older people are also able to use wearable and mobile devices. Also, the upper limit of age in the inclusion criteria was set to 75 years. Retrospectively this should have been set higher as we did receive interest from persons older than 80 years during the recruitment.

A total of 7590 symptom events were self-reported by patients, showing the potential for data collection. Just the symptom reports alone, or their number, may be useful, at least longitudinally. As an example, patients with DBS in our study reported remarkably few non-motor symptoms. From the point of view of generalizability, however, it is good to understand that events will not be evenly distributed across patients, so the effective number of patients becomes smaller than the number of study participants. Combining the event reports with the accelerometer data from a commercially available device opens possibilities for understanding symptoms and improving medication and overall care.

Data collection for supervised models requires a ground truth for the existence and severity of symptoms. Such studies, as opposed to monitoring when models already exist, require stricter design and can be more cumbersome for patients. The temporal accuracy of symptom diaries, for example, is critical, as is the accuracy of symptom evaluations. The latter is known to be difficult, even with videotaping and training [8,22].

If the goal is to contrast symptomatic and asymptomatic periods in the training of symptom-detection models, it is not clear what the control should be. For example, movements do not usually occur during sleep, and because we do not want the models to predict arousal states, sleep should be detected and removed, or arousal should be controlled in the models. But sleep is not well-defined, arousal not easy to measure, and the question about other potential confounders arises, including technical ones, such as willingness to wear the device, and data quality more generally. Using unsupervised models to pre-select movement states does not solve these issues.

The modeling pipeline for symptom quantification was relatively simple, power spectra of blocks followed by linear models or ´generalized linear models (GLM). Symptom types had characteristic spectral shapes that mostly replicated across data sets, and, from our own data collection, rather broad temporal envelopes, indicating either long-duration symptoms or low temporal resolution of symptom diaries. No clear advantage was shown by any of the accelerometer channels, including our derived virtual channels (except for low tremor amplitude in the direction of the limb). We did not attempt discriminative symptom detection.

In our analyses, data apart from VAEs was in blocks of 21.8 minutes as it was assessed to be compatible with the time scale of the symptom diaries. When symptoms are dynamically detected from raw data, much shorter intervals would be beneficial. Spectral resolution would be sufficient even in ten-second blocks, the temporal scale used with the VAE.

As seen by the simple models from accelerometer signal, MJFF Levodopa study shows attenuation of bradykinetic and amplification of dyskinetic signals in some fine-motor tasks, and bradykinesia also while walking. These and other movement states present in the MJFF experiment are detectable from unsupervised representations created with VAEs, and detection ability of the models remains across data sets. Further study is needed on combining movement-state detection by VAEs with actual symptom detection.

The primary objective of the study was the identification of OFF symptoms, as perceived by the subject, with reasonable accuracy from real-world data collected with commercially available Garmin Vivosmart 4 wearable device and a mobile application. With motor OFF symptoms, our findings are promising. Obviously more research with larger populations and improved methods in collecting the data is needed. While we did not demonstrate the potential of the full approach, we believe that with more patient data, one can train models to detect useful movement states, and then use supervised models or other ways to assess symptoms from chosen periods. This could provide some benefits of controlled measurement without its costs, inconvenience, and compliance issues. Model training may require a data collection plan that has both uncontrolled and controlled parts. Generalizability to clinical populations requires a larger patient sample than what was available in this research [23].

## Supporting information

S1 Fig 80% confidence intervals of the coefficients of the models predicting observer scores from accelerometer data. (TIF)

S2 Fig Drug effect on the tremor symptom severity as predicted from the accelerometer data (x-axis), and relative strength of the effect (y-axis) at different tasks of the MJFF data. (TIF)

S3 Fig Drug effect on the dyskinesia symptom severity as predicted from the accelerometer data (x-axis), and relative strength of the effect (y-axis) at different tasks of the MJFF data. (TIF)

S4 Fig Drug effect on the bradykinesia symptom severity as seen by observers (x-axis), and relative strength of the effect (y-axis) at different tasks of the MJFF data. (TIF)

S5 Fig Drug effect on the dyskinesia symptom severity as seen by observers (x-axis), and relative strength of the effect (y-axis) at different tasks of the MJFF data. (TIF)

S6 Fig Drug effect on the tremor symptom severity as seen by observers (x-axis), and relative strength of the effect (y-axis) at different tasks of the MJFF data. (TIF)
