# Supplementary figures and images for: Feasibility and patient acceptability of a commercially available wearable and a smart phone application in identification of motor states in parkinson’s disease
Source: PLOS Digit Health. 2023 Apr 7;2(4):e0000225. doi: 10.1371/journal.pdig.0000225 (PMC10081770; doi:10.1371/journal.pdig.0000225)

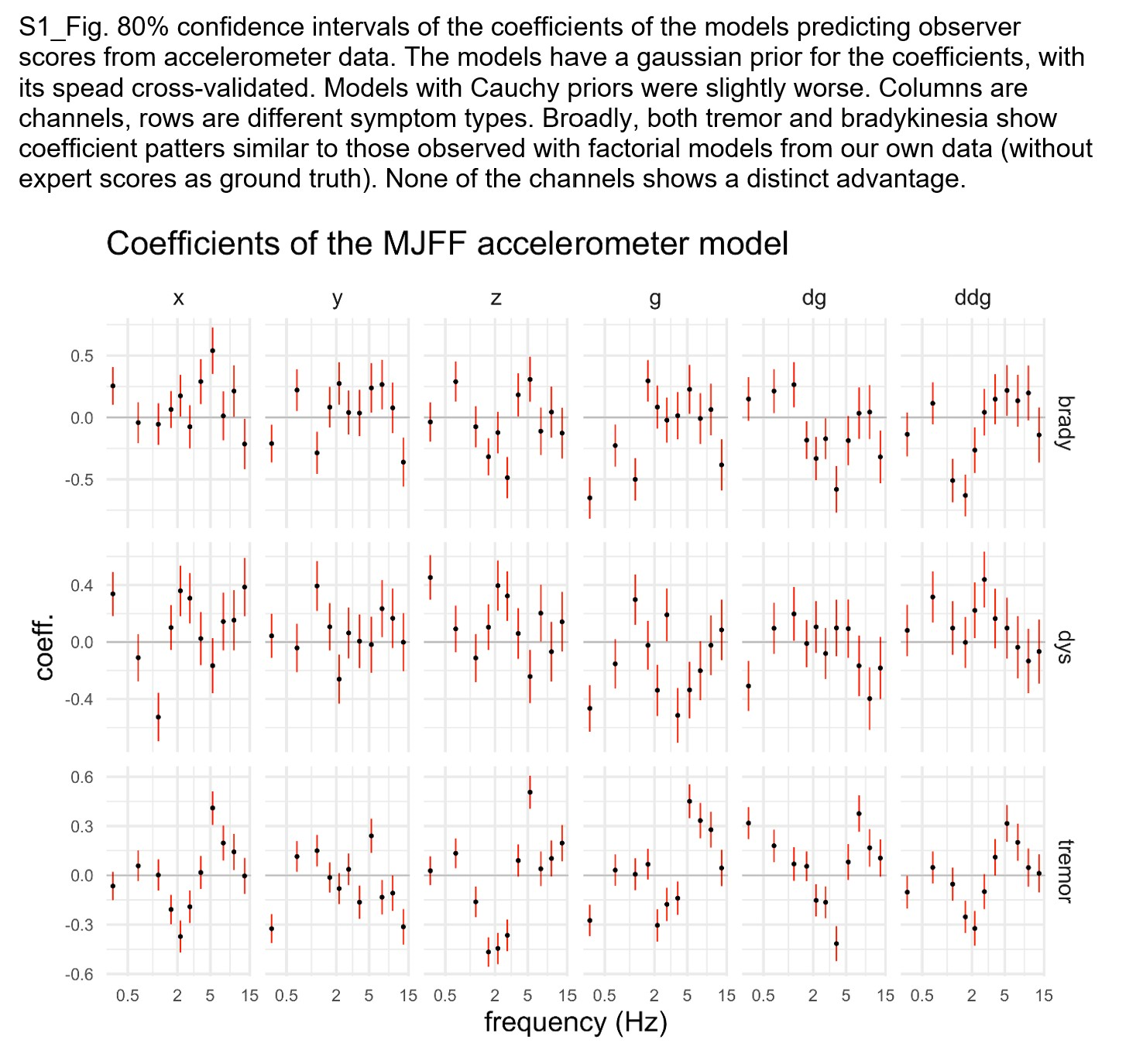

Supplement: S1 Fig — (TIF) [file pdig.0000225.s001.tif]

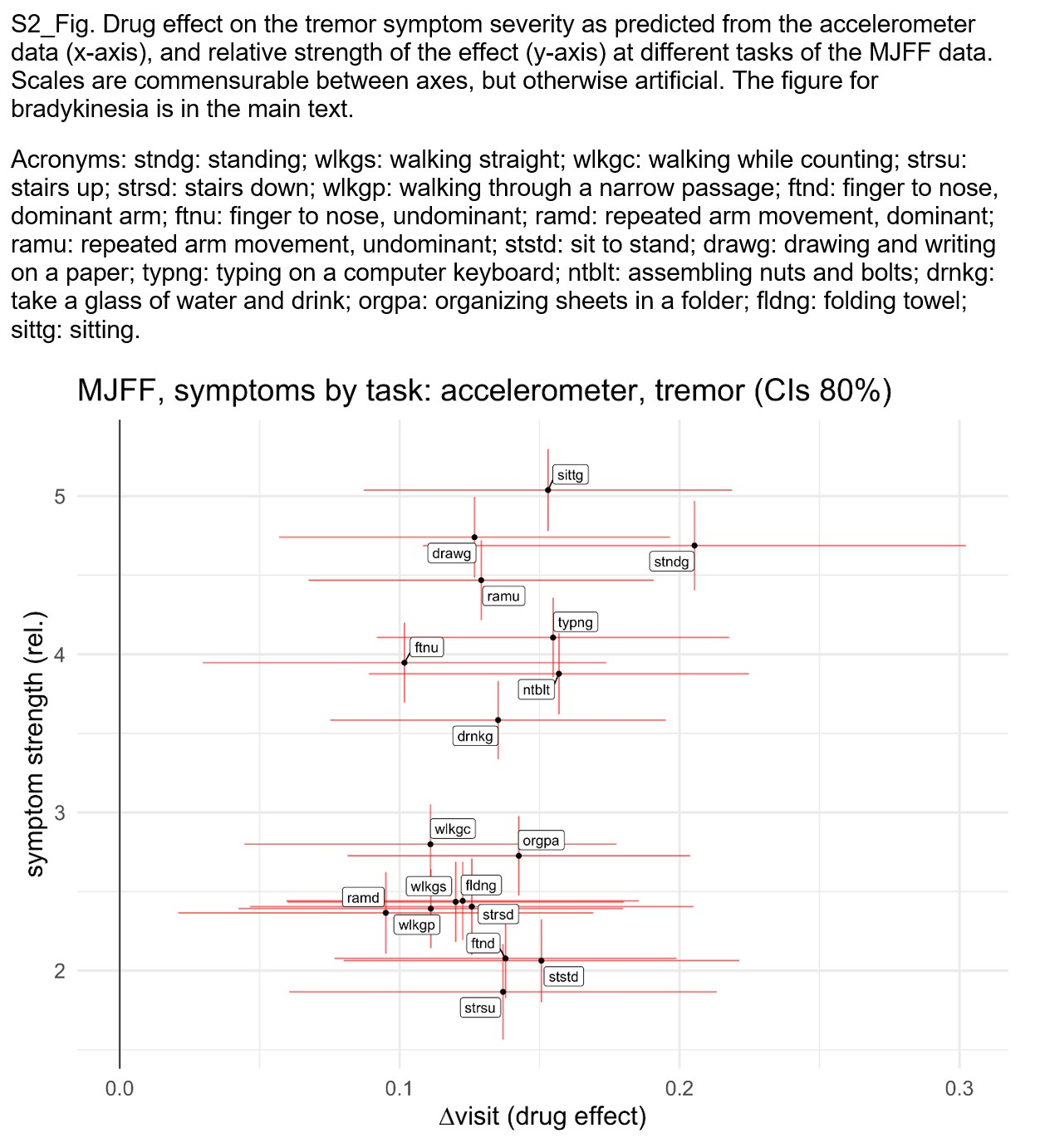

Supplement: S2 Fig — (TIF) [file pdig.0000225.s002.tif]

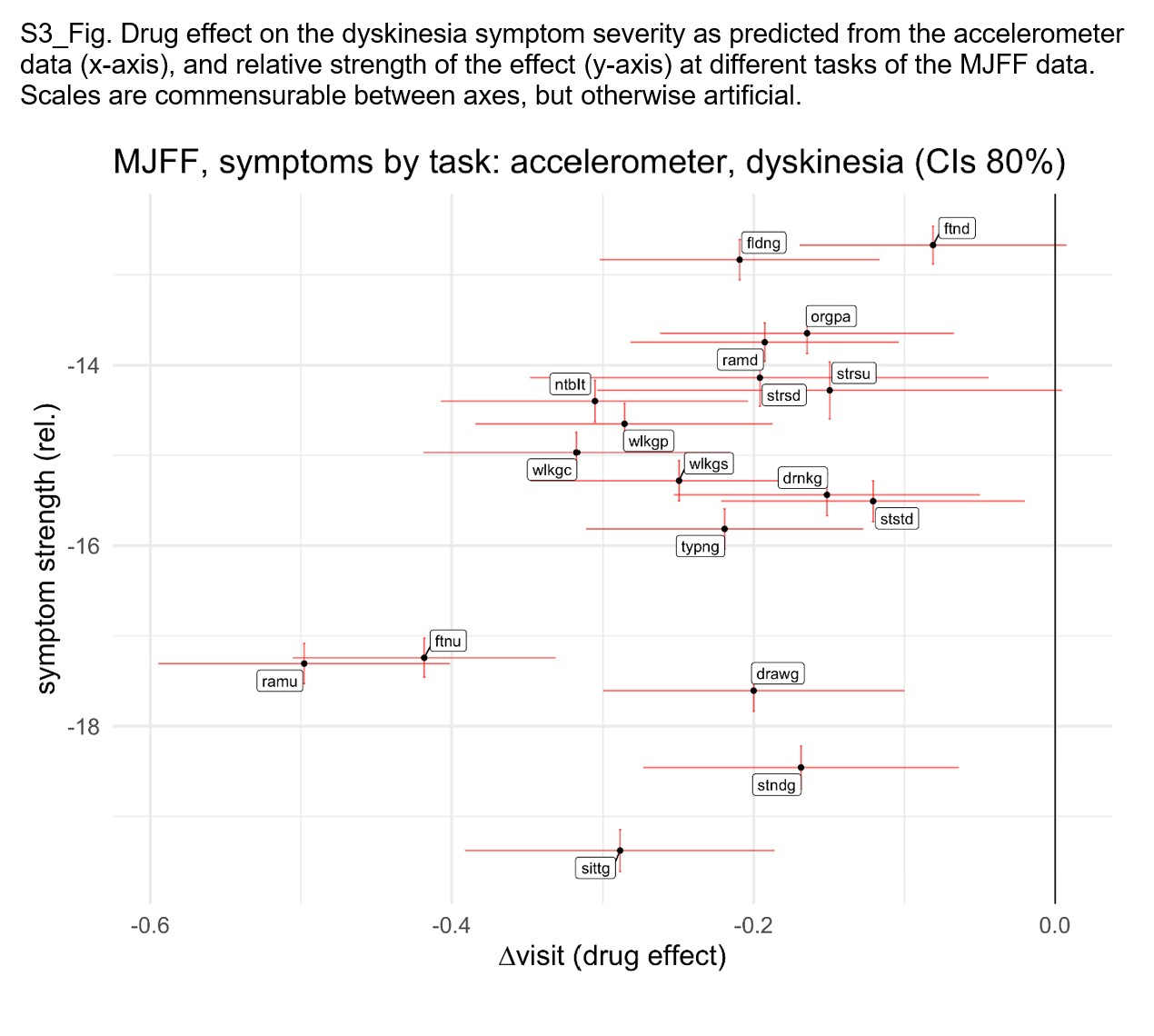

Supplement: S3 Fig — (TIF) [file pdig.0000225.s003.tif]

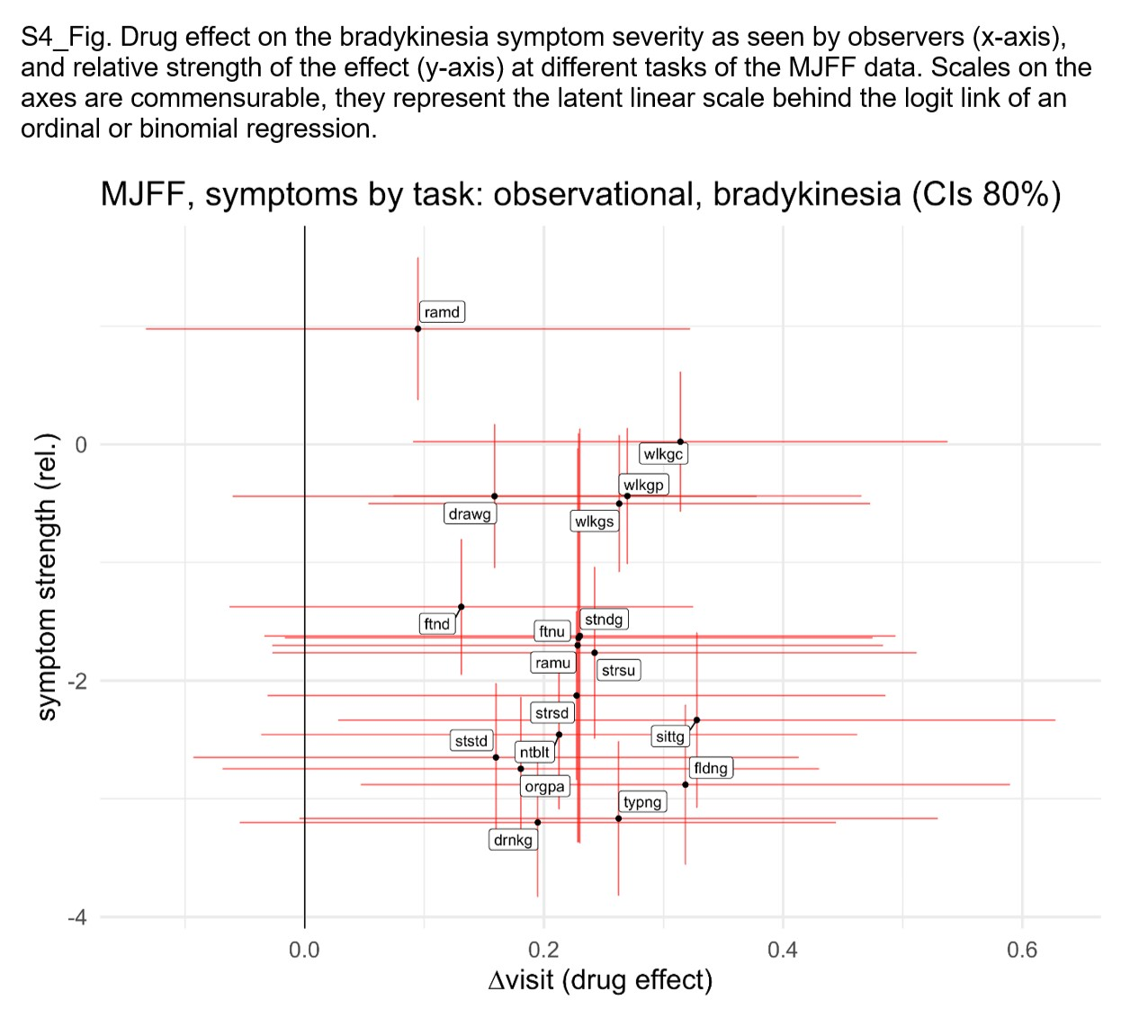

Supplement: S4 Fig — (TIF) [file pdig.0000225.s004.tif]

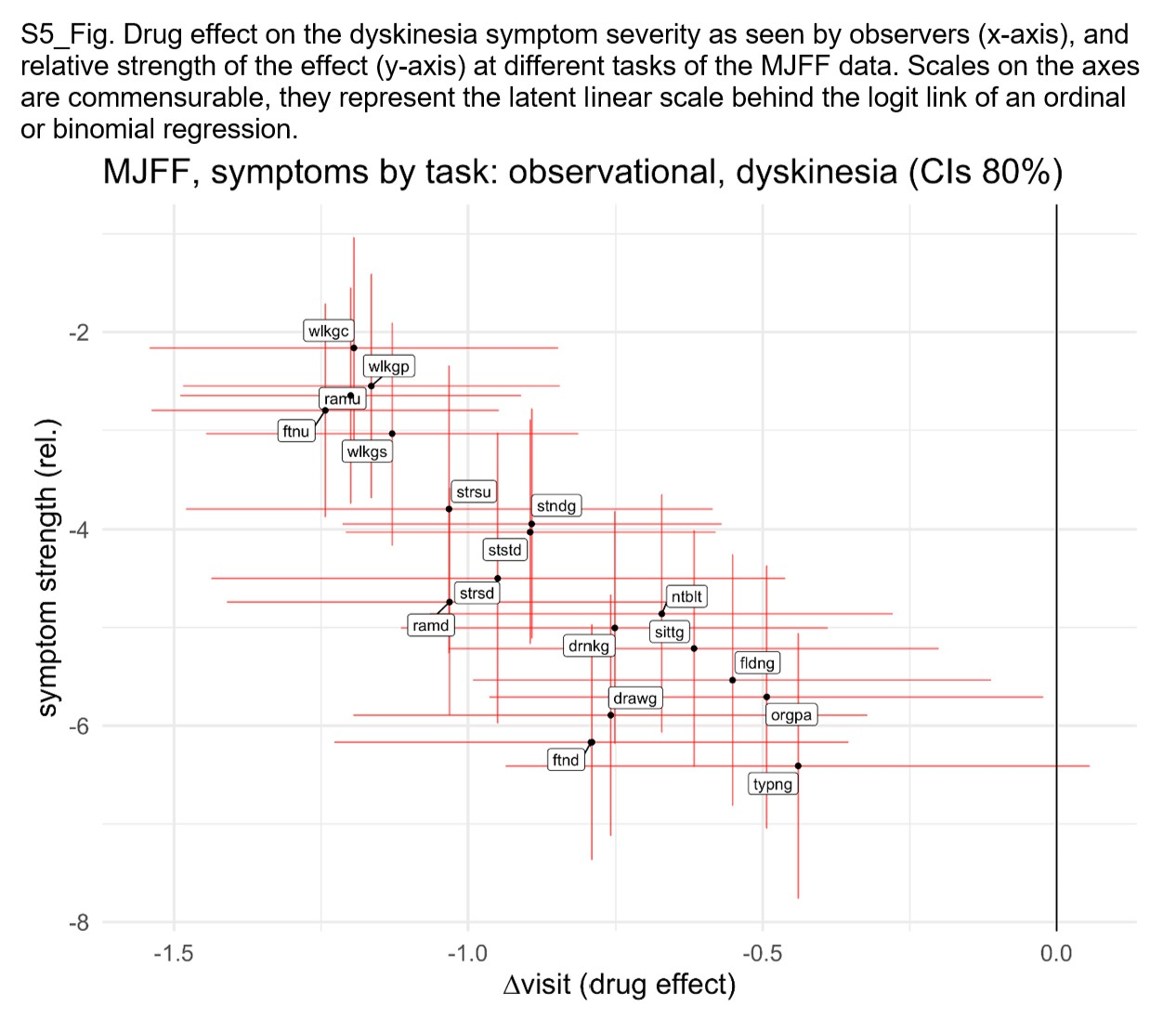

Supplement: S5 Fig — (TIF) [file pdig.0000225.s005.tif]

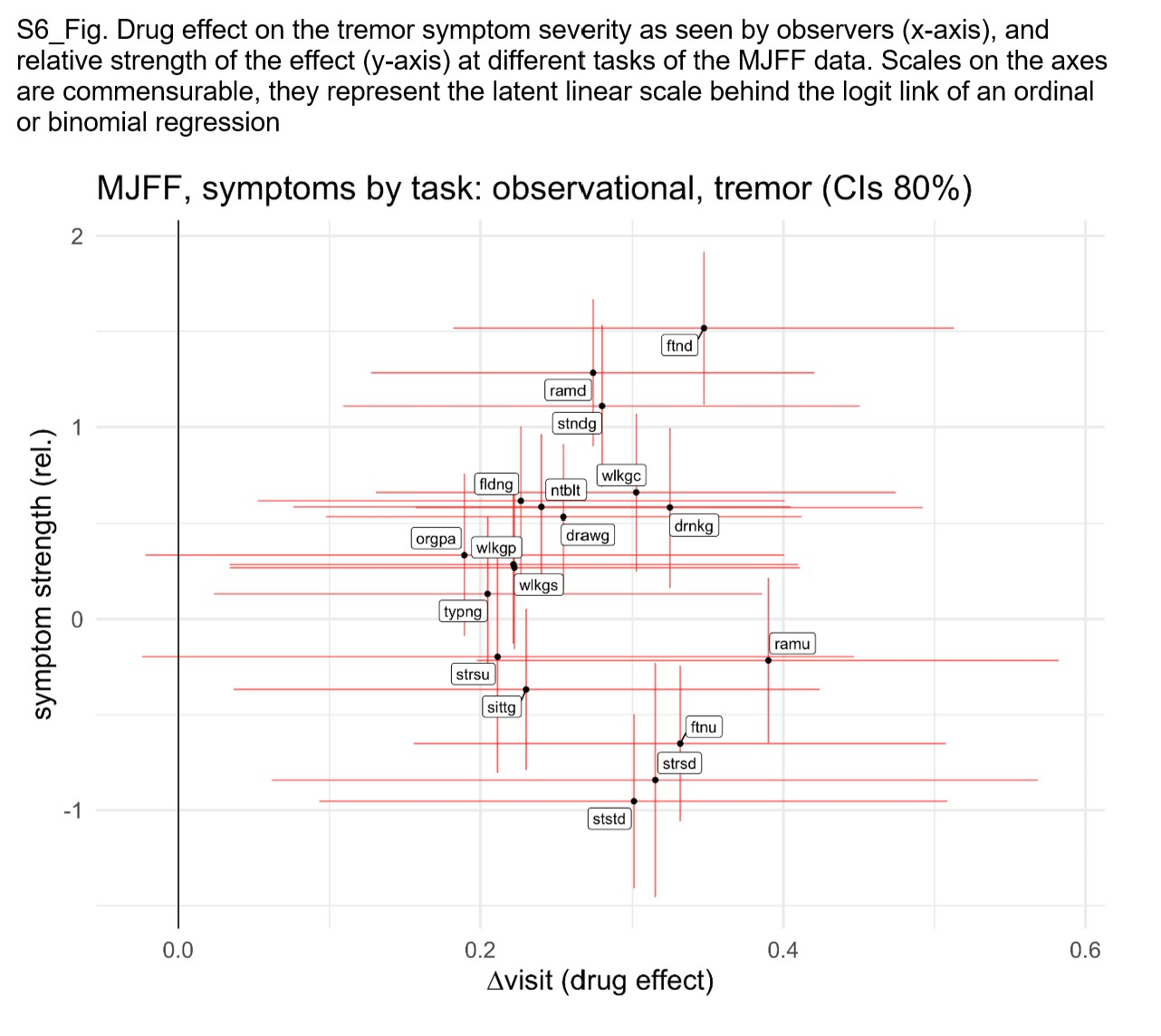

Supplement: S6 Fig — (TIF) [file pdig.0000225.s006.tif]
